# Non-invasive brain stimulation in information systems research: A proof-of-concept study

**DOI:** 10.1371/journal.pone.0201128

**Published:** 2018-07-26

**Authors:** Laurence Dumont, Félix Larochelle-Brunet, Hugo Théoret, René Riedl, Sylvain Sénécal, Pierre-Majorique Léger

**Affiliations:** 1 Psychology Department, Université de Montréal, Montréal, Canada; 2 Tech3Lab, HEC Montréal, Montréal, Canada; 3 Department of Business Informatics – Information Engineering, University of Linz, Linz, Austria; 4 Digital Business, School of Management, University of Applied Sciences Upper Austria, Steyr, Austria; University of Ottawa, CANADA

## Abstract

One of the founding experiments in the field of Neuro-Information-Systems (NeuroIS), which aims at exploring the neural correlates of the technology acceptance model, suggests that perceived ease of use (PEoU) is associated with activity in the dorsolateral prefrontal cortex (DLPFC) while perceived usefulness is associated with activity in the insula, caudate nucleus and anterior cingulate cortex. To further assess the link between DLPFC and PEoU, transcranial direct current stimulation (tDCS) was applied over bilateral DLPFC (F3 and F4) immediately before an online shopping task. Forty-two participants were divided in three stimulation groups: left anodal/right cathodal, left cathodal/right anodal and sham. No change in PEoU was observed post stimulation but participants in the left anodal/right cathodal stimulation group took longer to make a purchase compared to sham stimulation and had different visual fixation patterns over the buy buttons. This is, to our knowledge, the first use of non-invasive brain stimulation in the field of NeuroIS. Although the involvement of DLPFC in PEoU could not be confirmed, the present study suggests that non-invasive brain stimulation may be a useful research tool in NeuroIS.

## Introduction

In recent years, an increasing number of studies have been conducted in Information Systems (IS) research using neuroscience tools and theories. This emergent field of research, known as NeuroIS, aims to refine and better understand the cognitive and affective mechanisms underlying interactions with IT artifacts [1–4]. In one of the first NeuroIS studies, Dimoka and collaborators [5] investigated the neural correlates of the technology acceptance model (TAM) [6,7]. TAM aims to predict use and appreciation of technology using two main constructs, perceived ease of use (PEoU) and perceived usefulness (PU), which are usually measured using psychometric scales such as the WebQual instrument [8]. TAM was developed in the transition to the computer era in the 1980s and was initially used to evaluate the implementation of information systems in the workplace. It has since become a leading model in IS research and has been applied to contexts and domains as wide as software adoption in the workplace [9], user experience research [10] and marketing [11]. Using functional magnetic resonance imaging (fMRI) in 6 healthy adults, Dimoka and colleagues found that PEoU of a commercial website was associated with activity in the dorsolateral prefrontal cortex (DLPFC) whereas assessing PU recruited the caudate nucleus, anterior cingulate cortex and insula [5].

This pilot study showed that TAM constructs could be investigated with imaging technologies, but due to the fact that many cognitive functions are associated with each of the identified brain regions, the specific cognitive functions associated with the activated areas could only be hypothetical and not directly confirmed. It was suggested that PEoU effects were linked to cognitive effort and working memory (DLPFC) while PU was associated with utility (caudate nucleus, cingulate cortex) and evaluation of potential loss (insula)[5]. However, these structures are involved in other cognitive functions that could contribute to a task as complex as navigating and evaluating a web interface. For example, the DLPFC has been linked to working memory [12], but also to cognitive control [13], attentional bias [14], inhibition [15] and planning [16], to name just a few, all of which could potentially contribute to the PEoU and PU of an IT interface.

This issue has been discussed in a theoretical framework which categorizes experimental designs in applied neuroscience as able to state that a region is either associated with a concept, necessary or sufficient to explain this concept [17]. In that regard, previous evidence [5] associated PEoU and the DLPFC, whereas there are ways to determine whether the DLPFC is necessary or sufficient to explain PEoU. In light of this unresolved issue, it is necessary to confirm and expand the Dimoka et al. (2011) localization findings before addressing the more complex question of identifying the cognitive underpinnings of TAM.

Transcranial direct current stimulation (tDCS; [18,19]), a non-invasive brain stimulation (NIBS) technique, offers the possibility of establishing a causal relationship between brain and behavior. tDCS works on the premise that a weak constant current applied to the surface of the head through two surface electrodes of different polarity can modulate the resting membrane potential of the neurons it reaches transcranially. In turn, sustained modulation of membrane potentials can increase or reduce cortical excitability in surface brain areas, resulting in modified behavioral output. In the classical way of performing tDCS stimulation on a human participant, a secure and controlled 8 volts current source is equipped with two wires and electrodes. The electrodes are fitted to saline soaked sponges, generally ranging from 4 to 6 square inches. From that point, the current flows from the anode to the cathode through the different tissues in between. A low amperage, usually below 2 mA, is applied and continuously monitored by the tDCS device to insure constant and safe stimulation (see [19–24] for detailed explanations of the method.

When it is applied over the DLPFC, bilateral tDCS (where one electrode is placed over the left DLPFC and the other over the right DLPFC) was found to modulate behavioral output of cognitive functions such as risk taking [25,26], craving [27], decision making [28–30], emotion processing [31], emotional regulation [32], mental flexibility [33], language comprehension [34], learning [35], verbal performance [36], attention [37] and working memory [38].

The primary objective of this proof-of-concept study was to determine whether tDCS can be used to assess the contribution of specific brain areas to constructs used in research fields such as information systems and human-computer interaction. To this end, tDCS was applied over bilateral DLPFC to verify the existence of a causal relationship between DLPFC activity and PEoU of a commercial web site. Given the fact that no laterality hypothesis can be derived from the Dimoka and collaborators (2011) fMRI study, a bilateral stimulation protocol has the advantage of stimulating both DLPFC simultaneously with different polarities. This approach may thus maximize potential effects by interfering with both targets. By targeting and modulating DLPFC activity, we sought to determine whether this area plays a significant role in human interactions with user interfaces and whether tDCS can modify their subjective perception.

Based on the *a priori* hypothesis of a direct link between DLPFC and PEoU, as evidenced by fMRI [5], it is first hypothesized that bilateral tDCS over DLPFC will affect PEoU of a commercial website, and that it should not affect PU given the non-overlapping neural correlates that were previously identified. Given the reported effects of DLPFC tDCS on a wide variety of cognitive domains [39] and on EEG activity [40,41], it is also hypothesized that behaviors reflecting other cognitive and affective processes will be affected by stimulation. Such behaviors would be those directly involved in the completion of the goal of the task, namely time before purchase, fixation on the buy buttons and EEG activity in the seconds prior to the choice. Directional hypothesis on such processes are hard to make given the sometimes contradicting or inconclusive results of meta-analysis of tDCS effects over DLPFC [42,43]. Given the absence of specific research combining decision-making, tDCS over the DLPFC and EEG measures, analysis of the EEG data is exploratory.

## Method

### Sample and procedure

The experiment consisted of six visits to an online music store, during which participants had to choose and purchase one song of their liking in each visit, using a prepaid credit card. Each of the 6 visits lasted a maximum of 6 minutes to achieve comparable experimental length between visits and participants.

**Fig 1:**
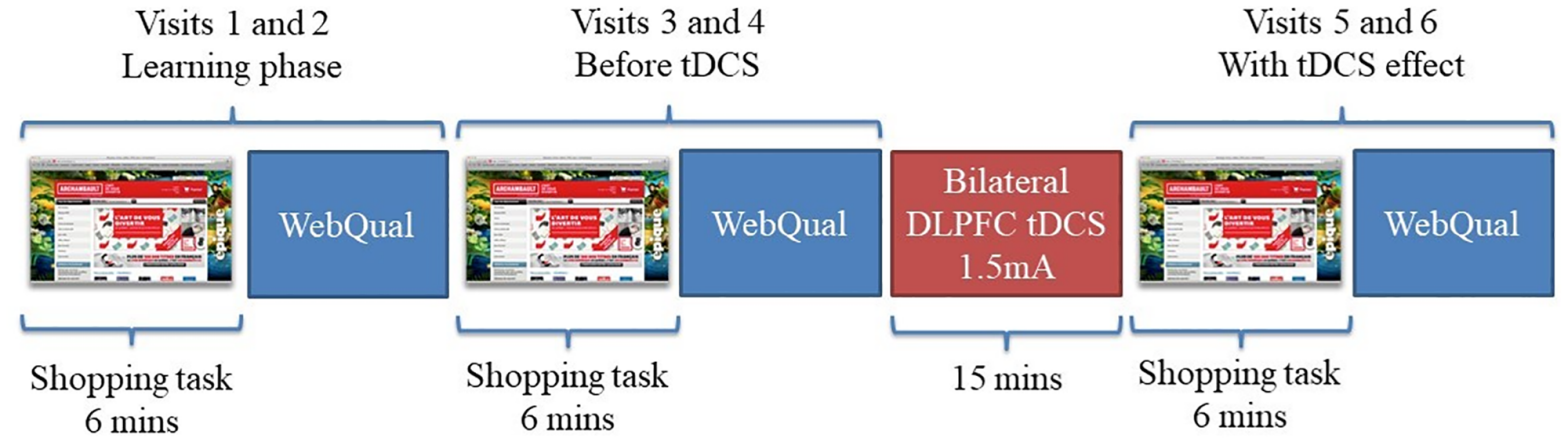
Experimental procedure.

As illustrated in Fig 1, two sets of two visits were made to the website before the tDCS stimulation session. Because online and offline tDCS have been shown to have a significant impact on learning and working memory [35,44], tDCS was applied when the tested behavior had completed its learning phase. To achieve this, the first two visits allowed participants to familiarize with the website, the third and fourth visits allowed a pre-stimulation measure and the last two visits were done after the tDCS protocol ended. After each visit, participants completed the Webqual questionnaire to assess PEoU and PU [8]. This questionnaire also includes items assessing the entertainment factor of the website.

A two factors experimental design was used, with stimulation condition as a between-subjects factor and pre- and post-stimulation behavior as a within-subjects factor. Forty-two participants were recruited and separated in three experimental groups (N = 14 in each group). The first group received left anodal/right cathodal stimulation, the second received left cathodal/right anodal stimulation, and the third group received sham stimulation (see Table 1).

**Table 1:** Sample description.

Stimulation Group	N (females)	Age (std.dev.)
Left anodal / right cathodal	14 (9)	22.41 (2.64)
Left cathodal / right anodal	14 (5)	23.46 (2.84)
Sham stimulation (placebo)	14 (10)	23.8 (3.01)

Electroencephalogram (EEG), eye-tracking data, and mouse/keyboard actions were simultaneously recorded on two different computers (one for EEG and the other for eye-tracking and user-generated actions) and were synchronized using a Noldus Synchbox (Wageningen, Netherlands), with a final delay shorter than 10 milliseconds between datasets. During tDCS stimulation, all recordings were stopped because of electrical current interference and participants were told to relax during stimulation. Data collection was resumed after stimulation and participants completed the last two visits and questionnaires. A short debriefing procedure took place after the experiment.

All participants were right handed and were between 18 and 35 years old. Further information on the samples can be found in Table 1. Upon contact by the research team, potential participants received a copy of the consent form, which they signed before the start of the experimental session Each participant received a 30$ Amazon gift certificate as compensation for participation and could also keep the products they had bought during the experiment (which ensured that participants took the task seriously since they went through a complete and real purchase procedure). This project was approved by the ethics research board (CERFAS) of the University of Montréal (13-115-CERES-D). Participants had to conform to the following exclusion criteria: 1) psychiatric or neurological disorder history, 2) history of head trauma resulting in loss of consciousness, 3) presence of a cardiac pacemaker, 4) presence of a piece of metal on the skull, 5) presence of tinnitus, 6) fainting history, 7) epileptic history, and 8) substance abuse.

### Experimental stimuli

The website used for the experiment was Zik.ca, a major online music store in Canada at the time of data collection. The experiment was conducted without contact with this company and results of the study will not directly serve, nor are they motivated or funded by, private company interests. Three IS experts evaluated the website using the WebQual measurement scale [8] to provide a general baseline outside of the tDCS experimental setting. It also allowed to make sure there would be no floor or ceiling effects in the ratings. Descriptive results for the present sample and experts are shown in Table 2 and a potential desirability bias can be observed between the experts and participants.

**Table 2:** Descriptive statistics of the WebQual subscales.

Sub-Scale	Visits	LA/RC		LC/RA		Sham		Experts	
		Average	Std.Dev	Average	Std.Dev	Average	Std.Dev	Average	Std.Dev
PEoU	Learning	6,253	0,927	6,768	1,152	6,167	1,176	4,711	0,823
	Pre-Stimulation	6,146	1,167	6,786	1,275	6,003	0,882		
	Post-Stimulation	6,173	1,025	6,783	1,250	6,211	0,914		
PU	Learning	6,155	0,871	5,220	0,960	4,863	0,891	5,778	0,609
	Pre-Stimulation	4,935	0,952	5,241	1,091	4,836	0,863		
	Post-Stimulation	5,021	0,994	5,247	1,020	5,015	0,708		

Note: LA/RC = Left anodal / right cathodal, LC/RA = Left cathodal / right anodal.

### Moments of interest in the task

Visits to the website were conducted in three groups of two, where the first two visits allowed participants to familiarize themselves with the interface, the third and fourth visits stabilized behavioral patterns and served as baseline performance, and the last two visits were used to evaluate the effects of tDCS. Each visit comprised two phases, before and after choosing the song. This moment was marked by a click on the “Buy” buttons, which was found next to the title of each item. The time between the beginning of the visit and the decision was computed using timing of clicks and URL changes in the Tobii data files. As it coincides with the final decision-making process, analysis of EEG and eye-tracking data was performed in the 30-second period preceding the purchase decision marked by pressing the “Buy” buttons.

## Material

### tDCS

A Magstim DC stimulator (Magstim, Whitland, UK) was used to deliver tDCS for 15 minutes at 1.5mA. The 5 by 7 cm sponge electrodes were placed on the left and right DLPFC, corresponding to the F3 and F4 sites of the International 10–20 EEG system as identified by the EEG headset. The current slowly increased in the first 30 seconds and slowly decreased during the last 30 seconds to minimize the tingling sensation sometimes associated with the beginning and end of stimulation. In the sham condition, the current ramp up was performed at the beginning of stimulation and was automatically turned off for the remaining stimulation period.

After completion of the fourth visit, the sponge electrodes were quickly slid under the inactive EEG headset guided by the position of the F3 and F4 electrodes and removed once the stimulation protocol was over. The position and impedance of the EEG headset was checked before resuming data collection for the fifth and sixth visits.

### Electroencephalography

EEG was recorded using EGI 32 electrodes nets (Eugene, Oregon) sampled at 512 hZ and analysed using Brain Vision software. A 60Hz notch filter was applied to remove electrical noise from the signal. Data in the 30 second window previous to the “Buy” decision were segmented out of the complete dataset and checked for artifacts using an independent component analysis. After a baseline correction, a Fast Fourier transform was performed to extract spectral power of the Delta (< 4Hz), Theta (4 to 7 Hz), Alpha (8 to 15 Hz) and Beta (16 to 31 Hz) bands time window of interest.

### Eyetracking

A Tobii X60 (Danderyd, Sweden) system was used to monitor eye movements and pupil dilatation, the threshold to detect a fixation was set at 200ms. All URL that had “Buy” buttons were grouped depending on the number of songs presented in a given page (which was between 1 and 10 buttons per webpage) and areas of interest (AOI) were drawn as a 80 pixel wide vertical column with a length depending on the number of songs presented on each webpage.

### Psychometric measures

PU and PEoU were measured using the WebQual scale, assessing the following website dimensions: 1) information quality, 2) interaction, 3) trust, 4) response time, 5) ease of understanding, 6) intuitive operations, 7) visual appeal, 8) innovativeness, and 9) emotional appeal. The dimensions 1 to 4 are used to estimate PU, and the dimensions 5 and 6 are part of the PEoU construct. The dimensions 7 to 9 are linked to the entertainment factor of the website.

### Statistical analysis

Repeated measures factorial ANOVAs were conducted on the main variables (PEoU and PU) and on all nine subscales of the WebQual, time between the start of the visit and “Buy” decision, and average fixation duration of the “Buy” buttons. *Visit* was the within subject factor and stimulation group was as a between subject factor. When necessary, simple effects tests and dependent, or independent, sample t-tests were conducted with Bonferonni corrections. The same type of ANOVA was also conducted on EEG data. Differences in spectral power for the four main frequency bands (Delta, Theta, Alpha and Beta) were compared for each visit and group at the two stimulation sites (F3 and F4).

To confirm that there were no baseline differences between groups, a series of one way ANOVAs were conducted on the pre-stimulation visits of each variable of interest and no significant differences were found (*p* > .075) except for the Response Time sub-scale of the WebQual (*p* = .024). One-way ANOVAs, followed by post-hoc Tukey tests when relevant, were also conducted to compare the percentage of change between the pre- and post-stimulation for each group. This further analysis allowed to see effects specific to the stimulation, without any influence from the learning that occurred in the first two visits.

## Results

### WebQual

**Fig 2:**
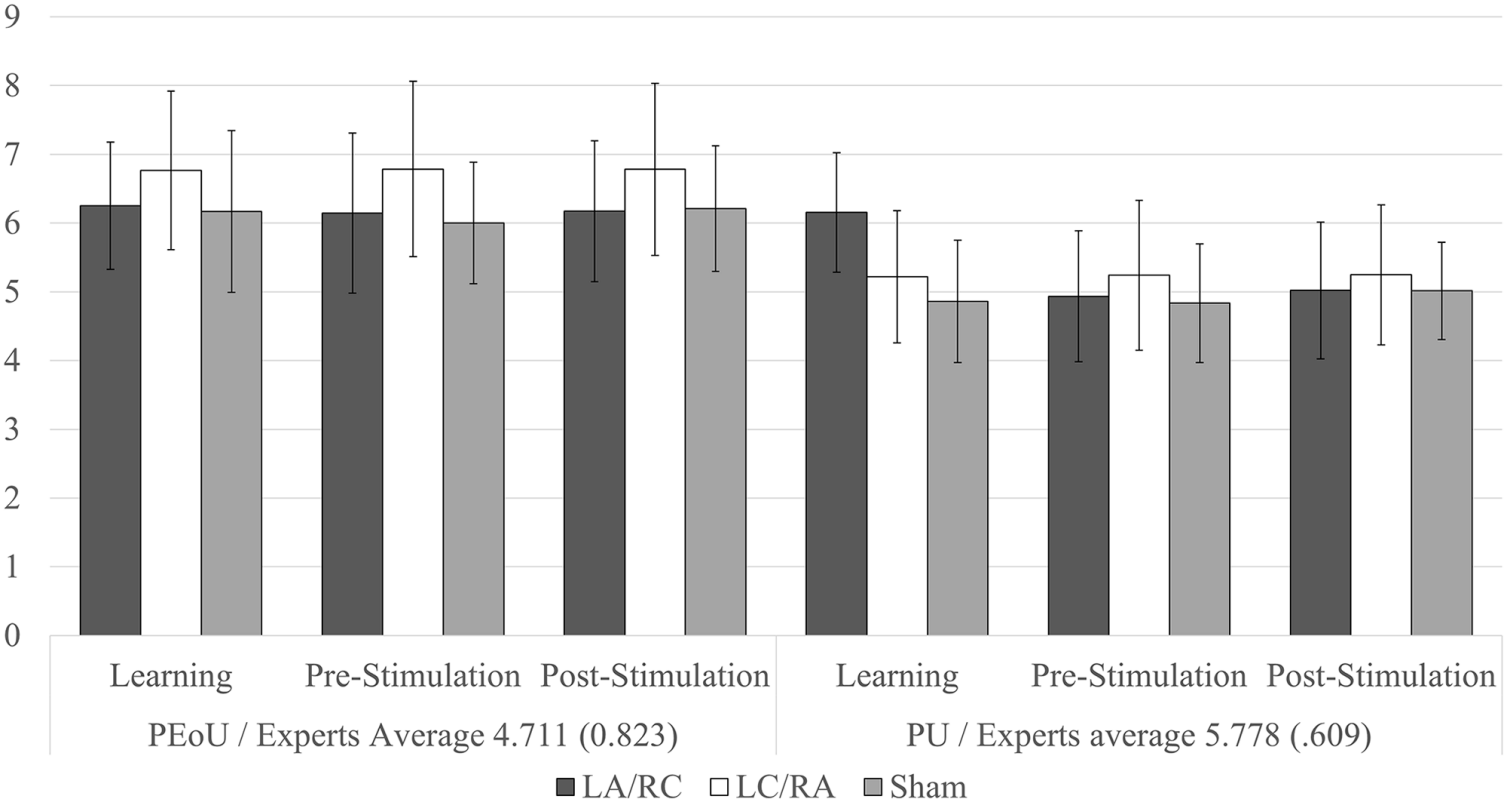
Perceived ease of use and perceived usefulness results. Expert evaluation values stated on the axis.

Assumptions of sphericity were violated in all scales and subscales of the WebQual (p ≤ .001). As a result, a Greenhouse-Geisser correction was applied to all results. Fig 2 shows results for PU and PEoU, in which there were neither effects of visit (*p* > .519), stimulation (*p* > .199) or interaction (*p* > .612). The only two significant observed results in any of the subscales (*p* < .05) were Emotive Appeal and Response Time.

**Fig 3:**
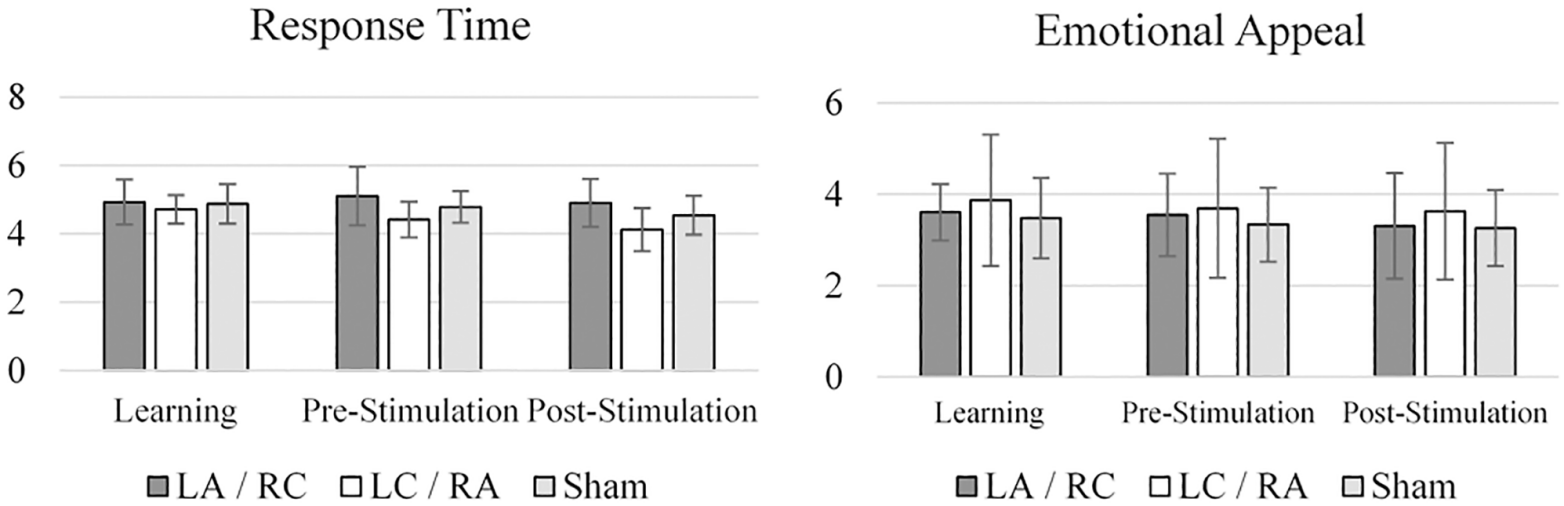
WebQual subscales with significant visit effects. LA/RC (Left anode / Right cathode); LC/RA (Left cathode / Right anode); Sham stimulation.

As shown in Fig 3, there were significant effects of *visit* (F(1.314) = 7.237; *p* = .004) and *group* (F(2) = 4.178; *p* = .023) in the subscale “Response Time” of PU construct, which assesses how fast and responsive the participant perceives the website. Simple effects analysis for the visit effect showed that scores were lower in the post-stimulation visits compared to the learning visits (*p* = .016) and the pre-stimulation visits (*p* = .02), but did not differ between learning and pre-stimulation visits (*p* = .811). As for the *group* effect, there was a significantly higher score (*p* = .019) for the left anodal / right cathodal group than in the right cathodal / left anodal group.

There was a significant effect of *visit* on the “Emotional Appeal” subscale (F(1.446) = 5.014; *p* = .018), where simple effects showed that scores significantly decreased between learning visits and post-stimulation visits (*p* = .041). For all other subscales, there were no group differences before (or after) the stimulation occurred.

### Time to purchase

**Fig 4:**
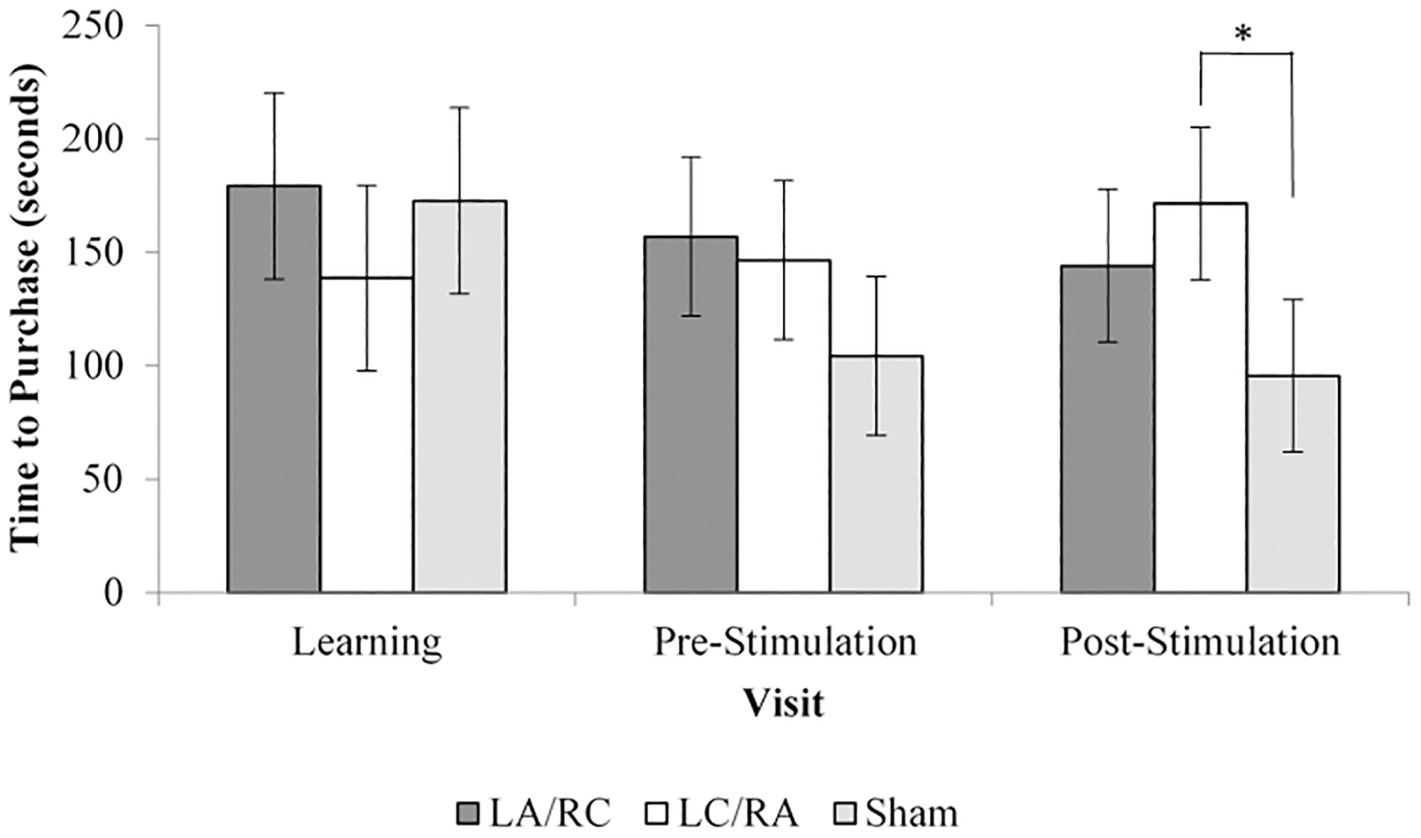
Time to purchase for each stimulation group and visit type. LA/RC (Left anode / Right cathode); LC/RA (Lect cathode / Right anode); Sham stimulation.

There was a main effect of *visit* (F(2) = 3.674; *p* = .03), no effect of *group* (F(2) = 1.82; *p* = .176) and the *interaction* between factors was significant (F(4) = 4.161; *p* = .004). Pairwise comparisons revealed no significant difference between visits (*p* > .06). 95% confidence intervals showed that the left cathodal / right anodal stimulation group took longer to select a song compared to the sham stimulation group in the post stimulation visits, as seen in Fig 4. Comparisons of percentage of change between the pre- and post-stimulation visits in Table 3 did not reveal significant differences between groups (F(2,39) = .252; *p* = .779) due to high variability in the direction of the effects of tDCS within groups, as seen in Table 4.

**Table 3:** Percentage of change in time to purchase before and after stimulation, by group.

Group	Mean	Standard deviation
Left anodal / right cathodal	12.86%	87.23%
Left cathodal / right anodal	33.63%	46.81%
Sham stimulation (placebo)	22.08%	90.86%

**Table 4:** Descriptive Statistics for the EEG power in the four main frequency bands for F3 and F4.

Frequency Band	Electrode	Group	Learning visits		Pre-Stimulation		Post-Stimulation	
			Average	SD	Average	SD	Average	SD
Alpha	F3	LA/RC	0.270	0.121	0.224	0.111	0.242	0.113
		LC/RA	0.317	0.108	0.260	0.101	0.246	0.139
		Sham	0.286	0.163	0.255	0.155	0.271	0.197
	F4	LA/RC	0.229	0.080	0.223	0.107	0.264	0.176
		LC/RA	0.349	0.183	0.287	0.122	0.250	0.091
		Sham	0.358	0.223	0.316	0.182	0.267	0.162
Beta	F3	LA/RC	0.261	0.242	0.167	0.146	0.154	0.090
		LC/RA	0.379	0.296	0.296	0.252	0.192	0.102
		Sham	0.223	0.313	0.142	0.083	0.172	0.149
	F4	LA/RC	0.238	0.188	0.227	0.213	0.340	0.612
		LC/RA	0.381	0.315	0.285	0.219	0.178	0.085
		Sham	0.265	0.244	0.258	0.185	0.175	0.100
Delta	F3	LA/RC	1.611	0.794	1.367	0.988	1.553	0.801
		LC/RA	1.920	1.259	1.770	1.232	1.366	0.961
		Sham	1.676	1.155	1.322	0.741	1.160	0.808
	F4	LA/RC	1.405	0.569	1.257	0.564	1.367	0.708
		LC/RA	1.897	1.454	1.579	0.733	1.330	0.765
		Sham	1.920	1.199	1.597	0.887	1.178	0.518
Theta	F3	LA/RC	0.534	0.241	0.476	0.235	0.477	0.155
		LC/RA	0.555	0.214	0.580	0.262	0.464	0.198
		Sham	0.490	0.208	0.454	0.145	0.400	0.138
	F4	LA/RC	0.455	0.169	0.428	0.188	0.405	0.151
		LC/RA	0.532	0.275	0.526	0.209	0.450	0.198
		Sham	0.565	0.216	0.515	0.180	0.452	0.185

### Fixation on the buy buttons

There was a main effect of *visit* (F(1) = 5.839; *p* = .022), no effect of *group* (F(2) = 0.727; *p* = .492) and the *interaction* between factors was significant (F(1) = 5.839; *p* = .022). Post hoc tests showed a decrease between pre-stimulation and post-stimulation visits for the left cathodal / right anodal (*p* = .012; -19.32%) and sham (*p* = .016; -22.12%) groups but not for the left anodal / right cathodal group (*p* = .234; +12.52%). When compared directly, the percentage of change between pre- and post-stimulation visits showed a significant group difference (F (37,2) = 4.45; p = 0.019), with Tukey post hoc tests congruent with the aforementioned effects.

#### EEG

Results of the EEG analysis can be found in Tables 5 and 6. Main effects of visit were found on F3 in the Beta range (p = .008) and on F4 in the Theta (p = .041) and Alpha ranges (p = .048). Linear contrasts showed a general linear decrease over time in all of these effects (p < .046). An interaction effect was found on F4 in the Beta range (p = .038). Table 6 shows that pre- and post-stimulation comparisons of power only showed significant differences in the Beta band in the F4 electrode. Post-hoc Tukey tests showed that Beta was in the Left-anode / Right-cathode group was significantly different from the decreases in the Left-cathode / Right-anode group (*p* = .036) and the sham group (*p* = .045), while these two decreases were not significantly different from each other (*p* = .987).

**Table 5:** ANOVA on EEG power on the F3 and F4 electrodes for visit and stimulation condition.

Electrode/Frequency band		Within Subject Effect of Visit			Between Subject Effect of Stimulation			Interaction Effect		
		Df	F	p	df	F	p	Df	F	P
F3	Delta	2, 56	1.151	0.324	2, 28	0.594	0.559	4, 56	0.246	0.907
	Theta	2, 56	1.545	0.222	2, 28	0.803	0.458	4, 56	0.431	0.785
	Alpha	2, 56	3.126	0.052	2, 28	0.491	0.617	4, 56	0.294	0.881
	Beta	2, 56	5.427	0.007	2, 28	1.099	0.347	4, 56	0.865	0.491
F4	Delta	2, 54	3.239	0.047	2, 27	0.309	0.737	4, 56	0.910	0.465
	Theta	2, 54	3.865	0.027	2, 27	0.717	0.497	4, 54	0.233	0.919
	Alpha	2, 54	4.834	0.012	2, 27	1.207	0.315	4, 54	1.603	0.201
	Beta	2, 54	1.224	0.302	2, 27	0.098	0.907	4, 54	2.524	0.05

**Table 6:** Percentages of change in EEG power between pre- and post-stimulation visits.

Location	Frequency band	Left anode / Right cathode		Left cathode / Right anode		Sham (placebo)		F	P
		Mean	Std. Dev	Mean	Std. Dev	Mean	Std. Dev		
F3	Delta	23.13%	59.18%	4.72%	83.32%	7.77%	75.74%	0.154	0.858
	Theta	22.09%	54.04%	-3.99%	51.09%	-7.66%	30.53%	1.165	0.326
	Alpha	16.02%	49.32%	11.29%	74.93%	9.74%	29.76%	0.033	0.967
	Beta	30.86%	78.94%	-6.24%	42.21%	19.63%	61.35%	0.973	0.390
F4	Delta	23.14%	36.12%	-12.95%	50.27%	-15.96%	45.34%	2.08	0.144
	Theta	12.95%	60.17%	-13.88%	23.83%	-10.78%	34.65%	1.211	0.313
	Alpha	31.95%	106.70%	-13.59%	21.99%	-10.76%	26.49%	1.756	0.191
	Beta	41.52%	83.64%	-22.90%	39.72%	-19.45%	35.29%	4.166	0.026

## Discussion

This study is the first, to our knowledge, to use tDCS to investigate the role of a specific brain structure in the interaction between a user and an IT artifact. Drawing upon the fMRI study of Dimoka et al. (2011), who found that DLPFC activity was associated with PEoU, this area was chosen as a target for tDCS to determine how modulating its excitability would affect the user in terms of technology acceptance and interaction behavior with a website. The present results do *not* support the hypothesis of a modulating effect of tDCS on PEoU, but provide partial support for the second hypothesis regarding the effects of tDCS on behavioral and neurophysiological aspects of the interaction with the website.

### Issues in tDCS

As mentioned earlier, there are conflicting results with regards to the efficacy of tDCS in modulating cognitive functions [39,42,43]. Of primary concern is recent data suggesting that tDCS is associated with very high levels of inter-subject variability. For example, some studies estimate that 45% of participants do not respond in the expected way to tDCS [45], which would be a facilitation of neuronal activity beneath the anode and the opposite beneath the cathode. Factors that can explain this response variability can be described in terms of stimulation parameters and individual differences [22]. As a result, a different stimulation protocol might have yielded significant results on PEoU, but since parameter space of tDCS is not well understood, it is difficult to make specific recommendations for such a protocol.

Nonetheless, many behavioral indicators were significantly affected by the stimulation protocol in the present study, which suggest that tDCS has the potential to influence processes involved in a complex task such as interacting with a website in an online shopping context. The EEG results are of particular relevance in this context, showing a physiological effect of the stimulation on a neurophysiological marker. This result substantiates the notion that the stimulation protocol affected brain activity, but that this effect was either not sufficient for our hypothesis to be supported or unrelated to our initial hypothesis.

### Methodological differences

Although it is difficult to directly compare data from techniques as different as fMRI and tDCS, some discrepancies in protocols between the present study and that of Dimoka and collaborators (2011) may partly explain conflicting results. For example, the experimental design used in the Dimoka et al. (2011) study compared interfaces that scored both very high and very low in terms of PEoU and PU. This may have elicited different processes than average-range websites such as the one used in this study. It is therefore possible that tDCS could modulate behavior when users are interacting with a web interface that is hard to use. Indeed, as state dependency theories of NIBS suggest [46], the efficacy of stimulation depends on the nature of the task and the neuronal activity in participant’s brain both before and during the task. For example, Benwell, Learmonth, Miniussi, Harvey, & Thut (2015)[47] found that effects of tDCS were only visible in a high workload task whereas there were no significant effect in an easier task.

A further distinction with the study of Dimoka and collaborators (2011) is the fact that brain activity was measured *while* participants recalled their interaction with the website rather than *during* the interaction in the fMRI study. This is in sharp contrast with the present study where behavioral and neurophysiological assessments were performed during the actual use of the website.

Another limitation of our results is the sensitivity of psychometric scales to manipulations in tDCS. tDCS effects are usually of a small magnitude [48,49] and a 7-point scale was probably not sensitive enough to provide robust effects. Furthermore, a recent meta-analysis showed that changes elicited by tDCS protocols usually affect reaction times in healthy participants and accuracy in clinical populations [43]. In the present study, significant changes were observed in decision time, which shows that the stimulation protocol had an effect on website use, which was probably too small to be detected by the WebQual questionnaire. Testing participants with very low proficiency in website use could provide an opportunity to assess this hypothesis.

### Involvement of the DLPFC in the interaction with a website

Interacting with a website in an online shopping context is a complex goal directed task which involves a multitude of cognitive function, some of which were brought forward by this experiment. This stresses the idea that specific cognitive functions may be vulnerable to tDCS and result in behavioral changes during complex operations such as interactions with a transactional website. The two main cognitive functions that could potentially be involved in that process are decision making and cognitive effort.

#### Behavioral indicators of decision making

The main result arguing in favor of an involvement of decision making processes in the interaction with a website is that eyetracking data (visual fixation) showed significantly reduced time spent on the buy buttons following left anodal/right cathodal stimulation, which was not the case for left cathodal / right anodal and sham stimulation. The buy buttons are visited when the decision-making process is in its later stages, so decreases in time spent by visual fixation following stimulation can indicate habituation in this part of the decision-making process for the sham and left cathodal /right anodal groups, since relevance of the information and content of this AOI stayed constant during the experiment. The absence of habituation in the left anodal /right cathodal group may indicate an impact of tDCS stimulation of the DLPFC on the efficacy of decision-making, which may be an underlying cognitive process behind PEoU. For example, an interaction with a website that is not designed to promote efficient decision making could make the website more difficult to use, hence affecting PEoU. Furthermore, left cathodal / right anodal stimulation of the DLPFC has been shown to promote conservative decision-making in healthy subjects [50], which is consistent with the present findings. It has also been shown to alter other aspects of decision making such as lying behavior [28,51], decision time [29] and confidence in decisions [30].

While post-hoc tests comparing the three post-stimulation groups showed increased decision time for the left cathodal/right anodal group compared to the placebo group, this effect disappeared when comparing percentage of change between the pre- and post-stimulation visits, suggesting that non-significant but slight pre-existing differences before stimulation may have been exacerbated following stimulation.

#### Neurophysiological indicators of cognitive effort

A decrease in Beta power at the F4 electrode was found in both active stimulation groups compared to sham, which suggests that tDCS had a significant effect on brain activity, which could be related to the behavioral changes found after the two active stimulation conditions, irrespective of polarity. The present data are in line with a previous study showing increased beta power following tDCS over the DLPFC. However, these effects were reported to occur *during*, but not *after*, stimulation (Song et al., 2014). Furthermore, it has been shown that bilateral stimulation of the DLPFC decreases frequencies above 15Hz (which include the Beta band) [52]. The beta band has mostly been associated with cognitive effort, working memory and visual attention [53] which could underlie some of the behavioral effects reported here. Taken together, the present data suggest that DLPFC activity may underlie the decision-making processes and cognitive effort associated with a goal-oriented interaction with a IS artefact. Additional studies are needed to disentangle the contribution of cognitive functions associated with the DLPFC to interaction with a website.

### Effects unrelated to tDCS

Some effects, which were not attributable to the stimulation protocol or to PEoU, were obtained in the WebQual scale and can be of interest to the IS community. The decrease in the emotional appeal of the website across visits may be attributed to a diminution of the novelty factor. It has long been known that repetition of a stimulus diminishes its affective value both in marketing, where this is known as the wear out effect [54], and in psychology [55]. Novelty is also seen as a critical dimension in the affective brain [56].

The decrease in the Response Time sub-scale suggests that participants find the website to be less responsive and fast as the study goes on. One of the possible explanations could be related to attentional understanding of time perception. To have an accurate perception of time at this scale, which requires attention and involves various loops between the basal ganglia, thalamus and related cortical structures, such that when attention is on other stimuli or processes, time can seem to go by faster [57]. When participants are still learning to interact with the interface, there are less resources to attribute to these neural signals, hence a perception of faster response of the website is to be expected.

## Conclusion

This study was a proof-of-concept of the use of tDCS in NeuroIS. Whereas no effect of DLPFC stimulation on PEoU ratings was observed, specific effects of stimulation were found on behavioral and neurophysiological measures during website interactions. Taken together, the present results suggest that tDCS may provide valuable insight into the cognitive mechanisms that underlie interaction with IT artifacts such as websites. This can only be achieved, however, if the neurophysiological effects of tDCS are better understood. Indeed, widespread use of tDCS in applied fields such as NeuroIS crucially depends on the establishment of stimulation parameter guidelines (intensity, duration, electrode size and placement, etc.) based on empirical evidence. Furthermore, before tDCS over areas such as the DLPFC can be used to assess the neuronal underpinnings of complex IS constructs (such as PU and PEoU, among others), its effects on cognitive functions such as decision-making, behavioral inhibition and working memory must be disentangled.
